# Numerical simulation for MHD Oldroyd-B fluid flow with melting and slip effect

**DOI:** 10.1038/s41598-024-58376-1

**Published:** 2024-05-08

**Authors:** Amit Dadheech, Surbhi Sharma, Qasem Al-Mdallal

**Affiliations:** 1 Department of Mathematics, Swami Keshvanand Institute of Technology, Management & Gramothan, Jaipur, India; 2 Department of Mathematical Sciences, UAE University, P.O. Box 15551, Al Ain, Abu Dhabi, United Arab Emirates

**Keywords:** Oldroyd-B fluid, Slip effect, Chemical reaction, Non-linear radiation, Engineering, Mathematics and computing

## Abstract

This investigation reflects an examination of Oldroyd-B fluid flow over a permeable surface subjected to the effects of melting, slip effect, inclined magnetic field and chemical reactions. The governing equations are resolved using the bvp4c inbuilt MATLAB tool, the arithmetic computation for the momentum, thermal and concentration equations are executed. The results are exhibited graphically. Numerical outcomes are graphically depicted by aid of velocity, concentration, temperature profiles for several model variables. The achieved results exhibit a promising agreement with the previously established findings available in the open literature. The results obtained indicated that Deborah number $\beta_{1}$ reduces the momentum boundary layer thickness whereas Deborah number $\beta_{2}$ enhances the adjacent momentum boundary layer. Furthermore, temperature profile declined for melting parameter $Me$. The application of this study transcends various engineering disciplines, offering practical solutions and optimization opportunities in polymer processing, coating technologies, cooling systems, materials processing, biomedical and environmental engineering.

## Introduction

Viscoelastic fluids, characterised by their combined elastic and viscous properties, have been the subject of extensive research across multiple scientific and engineering disciplines. Understanding their flow behavior is of paramount importance in numerous applications, such as polymer processing, food manufacturing, biomedical engineering, and oil industry operations. Among the various viscoelastic fluid, the Oldroyd-B fluid model stands out as a widely recognised and extensively studied model, offering insights into the unique characteristics of these complex fluids. Oldroyd-B model^1^ is defined by a constant-viscosity, incorporating both relaxation time and retardation time in its formulation. Hayat et al.^2^ illustrated the MHD BL flow for an Oldroyd-B fluid within a porous channel featuring suction/injection characteristics. Fetecau et al.^3^ suggested an unsteady flow of Oldroyd-B fluid over a plate. Tan et al.^4^ extended Stoke's first problem for the Oldroyd-B fluid within a porous medium, and they provided a precise solution by employing the Fourier Sine transform. Goyal and Sharma^5^ investigated the behavior of an Oldroyd-B fluid caused by an exponentially extending sheet, considering the influences of radiation and heat section/injection. Khan et al.^6^ explored the impact of heat generation 2D radiative effects on the flow of nanofluid caused by a nonlinearly stretchy surface with micro-organisms.

Studying the phenomenon of fluid flow and heat transfer in the presence of magnetic fields, known as magnetohydrodynamics (MHD), has gained significant attention due to its relevance in numerous scientific and engineering applications. In MHD boundary layer flow, a magnetic field applies a force to the charged particles present in the fluid, and this force has the potential to alter the fluid velocity, leading to the generation of vortices and turbulence. One particular problem that has received considerable interest is the MHD flow past a stretching sheet. This configuration represents a simplified model for a variety of practical situations, such as polymer processing, metal production, and boundary layer flows over solid surfaces. Numerous studies and advancements have emerged, contributing to a better understanding of fluid behavior in the presence of a magnetic field, as indicated by references^7–9^.

Heat transfer is a fundamental phenomenon that plays a crucial role in various fluid flow processes and engineering applications. Understanding and controlling heat transfer in fluid flows is essential for optimizing system performance, improving energy efficiency, and ensuring the reliability and safety of industrial processes. Fluid flow encompasses a wide range of applications including, but not limited to, thermal power generation, chemical processes, HVAC systems, and transportation. In these systems, heat transfer occurs through conduction, convection, and radiation, depending on the characteristics of the fluid, the surrounding environment, and the heat source or sink. Christov^10^ enhanced the model originally introduced by Maxwell–Cattaneo by reintroducing the partial derivative feature. As a result, the model that surfaced is widely acknowledged as the Cattaneo–Christov heat-flux model. In a study by Hosseinzadeh^11^, the flow of Maxwell fluid caused by a porous medium was examined, revealing that the Prandtl number exerts substantial influence on both the heat transfer coefficient and the fluid temperature. Gholinia et al.^12^ investigated the impact of thermal radiation on the flow of various nanofluids around a vertical cylinder. They noted that the fluid temperature increases due to the higher thermal conductivity of nanoparticles. Hashim et al.^13^ conducted a study on the hydro-magnetic nanofluid flow induced by a continuously enlarging sheet under convective boundary conditions at the sheet's surface.

The study of heat transfer during the melting process over stretched sheets represents a critical intersection of thermodynamics and fluid dynamics. The interaction between a solid surface and a moving fluid, coupled with the energy exchange accompanying the phase transition from solid to liquid, gives rise to a myriad of thermal complexities. Understanding the intricacies of melting heat transfer over a stretched sheet is crucial for optimising numerous industrial processes, such as polymer processing, crystal growth, and metal casting. Singh et al.^14^ explored the influence of melting-heat transport in the stagnation point flow of magnetohydrodynamic micro-polar fluid approaching a stretching sheet, employing carbon nanotubes in their investigation. Hayat et al.^15^ conducted a numerical examination to investigate the effects of melting heat transport and homogeneous-heterogeneous reactions in a flowing system. Following that, Epstein et al.^16^ and Ishak et al.^17^ inspected the melting-heat transfer in continuous laminar flow past a plate and a moving surface, respectively. Within a micropolar fluid context, Yacob et al.^18^ explored heat transfer in the BL stagnation-point flow toward a stretching sheet, incorporating the melting effect. Olkha et al.^19^ deliberated entropy analysis for the MHD flow over a stretchy sheet along with melting phenomena. Dadheech et al.^20^ studied MHD flow across a stretching surface for Casson fluid in the presence of melting and slip-effects. Dadheech et al.^21^ deliberated entropy optimization for the non-Newtonian fluid over a vertical plate along with Cattaneo-Christov heat flux and slip effect.

The slip effect, which allows for relative motion between the fluid and the solid surface, introduces a layer of complexity that significantly influences the overall behavior of the system. This condition has been recognized as a crucial factor in various engineering scenarios, including polymer processing, coating applications, and aerodynamics. The impact of slip-on fluid dynamics becomes particularly pronounced when studying the stretching sheet phenomenon, as the interplay between fluid flow and surface deformation can lead to fascinating and often counterintuitive outcomes. Labropulu et al.^22^ discoursed the effects of slip BC (boundary condition) for non-Newtonian fluid flows. Ali et al.^23^ examined slip phenomena in non-Newtonian viscoelastic fluid flow owing to oscillatory continuous stretched surface. Govindarajan et al.^24^ deliberated slip, mass-transport phenomena in a vertical channel in light of radiation. Dawar et al.^25^ investigated slip flow of a Maxwell fluid induced by a stretchable surface in non-linearly manner. Furthermore, it is noteworthy to mention that similar investigations have been conducted and documented in previous studies^26–41^, which are relevant to the current context.

The primary purpose of this study is to explore the flow behavior of an MHD Oldroyd-B fluid and its heat transfer characteristics over a stretching sheet, accounting for the presence of melting and slip effects, which have not been adequately explored in existing literature. Moreover, the study is observed in the presence of a porous medium, including melting and slip effects, adding novelty to our investigation. Significant implications of the showcased demonstration are outlined for application in engineering setups and the effective improvement of systems involving thermo-fluid flow, polymer processing, and similar domains. Various results related to physical parameters are explicitly explained.

## Mathematical formulation

**Figure 1:**
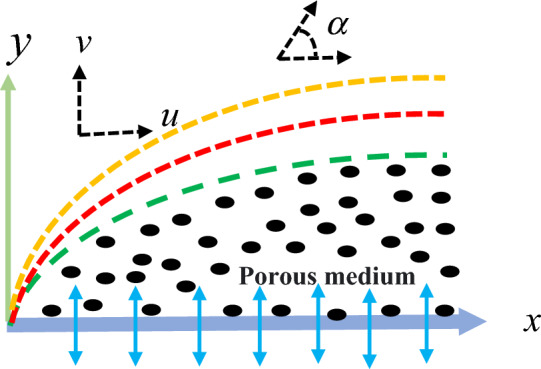
Physical model of the problem.

Here we considered a steady-state boundary layer flow, as well as heat and mass transfer, involving an incompressible Oldroyd-B fluid flow within a porous medium over a permeable surface, as illustrated in Fig. 1. The surface under consideration is undergoing linear stretching along the x-direction with a velocity that is described by $u_{w} = bx$, taking $b > 0$.

The governing system of equations for Oldroyd-B fluid, as proposed by Goyal and Sharma^5^, along with their respective boundary conditions (BC), are mentioned below:

1 $$\frac{\partial u}{{\partial x}} + \frac{\partial v}{{\partial y}} = 0\,\,$$

2 $$\begin{aligned} & u\frac{\partial u}{{\partial x}} + v\frac{\partial u}{{\partial y}} + \underbrace {{\lambda_{1} \left( {u^{2} \frac{{\partial^{2} u}}{{\partial x^{2} }} + v^{2} \frac{{\partial^{2} u}}{{\partial y^{2} }} + 2uv\frac{{\partial^{2} u}}{\partial x\partial y}} \right)}}_{Non - newtonian\,term} = \upsilon \frac{{\partial^{2} u}}{{\partial y^{2} }} \\ & \quad + \underbrace {{\lambda_{2} \upsilon \left( {u\frac{{\partial^{3} u}}{{\partial x\partial y^{2} }} + v\frac{{\partial^{3} u}}{{\partial y^{3} }} - \frac{\partial u}{{\partial y}}\frac{{\partial^{2} u}}{{\partial y^{2} }} - \frac{\partial u}{{\partial y}}\frac{{\partial^{2} v}}{{\partial y^{2} }}} \right)}}_{Non - newtonian\,term} - \underbrace {{\frac{{\sigma B_{0}^{2} \sin^{2} \alpha }}{\rho }\left( {u + \lambda_{1} v\frac{\partial u}{{\partial y}}} \right)}}_{Megnetic\,field} - \underbrace {{\frac{\upsilon }{{k_{p} }}u}}_{Porous\,medium\,term} \\ \end{aligned}$$

3 $$u\frac{\partial T}{{\partial x}} + v\frac{\partial T}{{\partial y}} = \frac{\kappa }{{\rho C_{p} }}\frac{{\partial^{2} T}}{{\partial y^{2} }} - \underbrace {{\frac{1}{{\rho C_{p} }}\frac{{\partial q_{r} }}{\partial y}}}_{Radiative\,term} + \frac{{\sigma B_{0}^{2} \sin^{2} \alpha }}{{\rho C_{p} }}u^{2} + \underbrace {{\frac{{q^{*} }}{{\rho C_{p} }}}}_{Heat\,source}$$

4 $$u\frac{\partial C}{{\partial x}} + v\frac{\partial C}{{\partial y}} = D_{m} \frac{{\partial^{2} C}}{{\partial y^{2} }} - \underbrace {{k_{n} (C - C_{\infty } )^{n} }}_{Chemical\,reaction}$$

To elucidate the physical parameters, kindly consult the provided nomenclature. A suitable boundary condition for the fluid flow, concentration and temperature (Olkha et al.^7^), is given by:

5 $$\begin{aligned} & at\,\,y = 0\,\,\,\,\left\{ {\begin{array}{*{20}l} {u = \underbrace {{u_{w} + L_{1} \frac{\partial u}{{\partial y}}}}_{Velocity\,\,Slip},\,\,\underbrace {{v = \kappa \frac{1}{{\rho \left\{ {\beta_{m} + c_{s} \left( {T_{m} - T_{0} } \right)} \right\}}}\frac{\partial T}{{\partial x}} - v_{m} }}_{melting\,\,term},} \hfill \\ {\underbrace {{T = T_{m} + L_{2} \frac{\partial T}{{\partial y}}}}_{Temperature\,\,Slip},} \hfill \\ {\underbrace {{C = C_{w} + L_{3} \frac{\partial C}{{\partial y}}}}_{Mass\,\,Slip},} \hfill \\ \end{array} } \right. \\ & at\,\,y \to \,\,\,\,\,\left\{ {\begin{array}{*{20}l} {u \to 0,\,\,} \hfill \\ {T \to T_{\infty } \,,\,\,} \hfill \\ {C \to C_{\infty } ,} \hfill \\ \end{array} } \right. \\ \end{aligned}$$

For radiative heat transfer, Rosseland diffusion flux model is considered. Following Siegel and Howell (1972) it reduces to following form

$$q_{r} = - \frac{{4\sigma_{1} }}{{3k^{*} }}\frac{{\partial T^{4} }}{\partial y}$$

Here it is supposed that the medium is optically thick and gray; absorbing-emitting, but non-scattering.

We assumed that the temperature variations within the fluid flow are significantly negligible. By expanding $T^{4}$ using Taylor's series around $T_{\infty }$ and neglecting higher order terms, we obtain:

$$T^{4} \approx 4T_{\infty }^{3} T - 3T_{\infty }^{4}$$

then

$$q_{r} = \frac{{ - 16\sigma_{1} T_{\infty }^{3} }}{{3k^{*} }}\frac{\partial T}{{\partial y}}$$

Were $\sigma_{1}$ and $k^{*}$ are the Stefan-Boltzmann constant and the mean absorption coefficient for radiation respectively.

### Solution

Implementing the similarity transformations listed below (Goyal et al.^5^):

6 $$\begin{gathered} \eta = y\sqrt{\frac{b}{v}} ,\,\,\,\,\,\,\,\,u = bxf^{\prime}(\eta ),\,\,\,\,\,\,\,\,\,\,v = - \sqrt {bv} f(\eta ),\,\,\,\,\,\,\,\, \hfill \\ \phi (\eta ) = \frac{{C - C_{\infty } }}{{C_{w} - C_{\infty } }},\,\,\,\,\,\,\,\,\,\,\theta (\eta ) = \frac{{T - T_{\infty } }}{{T_{m} - T_{\infty } }} \hfill \\ \end{gathered}$$

Continuity equation is identically self-satisfied. Employing Eq. (6) in (2–4) will result in following non-linear ODE’s:

7 $$\begin{aligned} & f^{\prime\prime\prime} - f^{{\prime}{2}} + f^{\prime\prime}\,f + \beta_{1} \left( {2f\,f^{\prime}\,f^{\prime\prime} - f\,^{2} f^{\prime\prime\prime}} \right) \\ & \quad + \beta_{2} \left( {f^{{\prime\prime}{2}} - ff^{iv} } \right) - \left\{ {M\sin^{2} \alpha \left( {f^{\prime} + \beta_{1} ff^{\prime\prime}} \right)} \right\} - Kp\,f^{\prime} = 0 \\ \end{aligned}$$

8 $$\begin{aligned} & \theta^{\prime\prime} + A^*f^{\prime} + B^*\theta + \frac{4}{3}R\left[ {\left\{ {(\theta_{w} - 1)\theta + 1} \right\}^{3} \theta ^{\prime\prime} + 3(\theta_{w} - 1)\left\{ {(\theta_{w} - 1)\theta + 1} \right\}^{2} \theta^{{\prime}{2}} } \right] \\ & \quad + \Pr \left( {Ec\,M\,\sin^{2} \alpha f^{{\prime}{2}} + f\theta ^{\prime}} \right) = 0 \\ \end{aligned}$$

9 $$\phi ^{\prime\prime} - Sc\,\left( {Kn\,\phi^{n} - f\phi ^{\prime}} \right) = 0$$

Transformed B.C. are:

BC

10 $$\begin{aligned} & a\,t\,\,\,\,\eta = 0\,\,\,\,\,\left\{ {\begin{array}{*{20}l} {f^{\prime}(\eta ) = 1 + \delta_{1} f^{\prime\prime}(\eta ),} \hfill \\ {\theta (\eta ) = 1 + \delta_{2} \theta^{\prime}(\eta ),} \hfill \\ {\,\phi (\eta ) = 1 + \delta_{3} \phi^{\prime}(\eta ),} \hfill \\ {f(\eta ) = S - \frac{Me}{{\Pr }}\theta^{\prime},} \hfill \\ \end{array} } \right. \\ & a\,s\,\,\eta \to \infty \,\,\,\left\{ {\begin{array}{*{20}l} {f^{\prime}(\eta ) \to 0,\,\,} \hfill \\ {\theta (\eta ) \to 0,} \hfill \\ {\phi (\eta ) \to 0,} \hfill \\ \end{array} } \right. \\ \end{aligned}$$

where Magnetic field parameter: $M = \frac{{\sigma B_{0}^{2} }}{\rho b}$, Prandtl number: $\Pr = \frac{{\mu C_{p} }}{\kappa }$, Schmidt number: $Sc = {\upsilon \mathord{\left/ {\vphantom {\upsilon {D_{m} }}} \right. \kern-0pt} {D_{m} }}$, Suction/Injection coefficient: $S = \frac{{V_{0} }}{{\sqrt {b\upsilon } }}\,$, Porosity parameter: $Kp = {\upsilon \mathord{\left/ {\vphantom {\upsilon {bk_{p} }}} \right. \kern-0pt} {bk_{p} }}$, Chemical reaction parameter: $Kn = {{k_{n} \left( {C_{w} - C_{\infty } } \right)} \mathord{\left/ {\vphantom {{k_{n} \left( {C_{w} - C_{\infty } } \right)} b}} \right. \kern-0pt} b}$, Velocity slip parameter: $\delta_{1} = L_{1} \sqrt {{b \mathord{\left/ {\vphantom {b \upsilon }} \right. \kern-0pt} \upsilon }}$, Melting surface parameter: $Me = \frac{{\left( {T_{m} - T_{\infty } } \right)C_{p} }}{{\beta_{m} + c_{s} \left( {T_{m} - T_{0} } \right)}}$, Temperature slip parameter: $\delta_{2} = L_{2} \sqrt {{b \mathord{\left/ {\vphantom {b \upsilon }} \right. \kern-0pt} \upsilon }}$, Radiation Parameter: $R = {{4\sigma_{1} T_{\infty }^{3} } \mathord{\left/ {\vphantom {{4\sigma_{1} T_{\infty }^{3} } {\kappa k^*}}} \right. \kern-0pt} {\kappa k^*}}$, Mass slip parameter: $\delta_{3} = L_{3} \sqrt {{b \mathord{\left/ {\vphantom {b \upsilon }} \right. \kern-0pt} \upsilon }}$, $\beta_{1} = \lambda_{1} c$ and $\beta_{2} = \lambda_{2} c$ are Deborah number in relaxation and retardation time respectively, $S = {{v_{w} } \mathord{\left/ {\vphantom {{v_{w} } {\sqrt {b\upsilon } }}} \right. \kern-0pt} {\sqrt {b\upsilon } }}$: suction/injection parameter.

### Methodology

Equations (7–9) are resolved using the bvp4c tool in MATLAB, following the boundary condition specified in (10). Initially, the nonlinear Eqs. (7–10) were expressed as a system of linear equations:

$$\begin{aligned} &f = y_{1} ,\,f^{\prime} = y_{2} ,\,f^{\prime\prime} = y_{3} ,\,f^{\prime\prime\prime} = y_{4} ,\,f^{\prime\prime\prime\prime} = y_{4}^{\prime } \hfill \\ &\theta = y_{5} ,\,\theta^{\prime} = y_{6} ,\,\theta^{\prime\prime} = y_{6}^{\prime } \hfill \\ &\phi = y_{7} ,\,\phi^{\prime} = y_{8} ,\,\phi^{\prime\prime} = y_{8}^{\prime } \hfill \\ \end{aligned}$$

$$y_{4}^{\prime } = \frac{{\left\{ \begin{gathered} y_{4} - y_{2}^{2} + y_{1} y_{3} + \beta_{1} \left( {2y_{1} y_{2} y_{3} - y_{1}^{2} y_{4} } \right) \hfill \\ \,\,\,\,\,\,\,\,\,\,\,\,\,\,\,\,\,\,\,\,\,\, + \beta_{2} y_{3}^{2} + \left\{ {M\sin^{2} \alpha \left( {y_{2} + \beta_{1} y_{1} y_{2} } \right)} \right\} + y_{2} Kp\, \hfill \\ \end{gathered} \right\}}}{{\beta_{2} y_{1} }}$$

$$y_{6}^{\prime } = \frac{{ - \left[ {A^*y_{2} + B^*y_{5} + 4R(\theta_{w} - 1)\left\{ {(\theta_{w} - 1)\theta + 1} \right\}^{2} y_{6}^{2} + \Pr \left( {Ec\,M\,\sin^{2} \alpha y_{2}^{2} + y_{1} y_{6} } \right)} \right]}}{{\left( {1 + \frac{4}{3}R\left\{ {(\theta_{w} - 1)\theta + 1} \right\}^{3} } \right)}}$$

$$y_{8}^{\prime } = Sc\,\left( {Kn\,y_{7}^{n} - y_{1} y_{8} } \right)$$

BC

$$\begin{aligned} & y_{2} (0) = 1 + \delta_{1} y_{3} (0),\,\,\,\,y_{1} (0) = S - \frac{Me}{{\Pr }}y_{6} (0),\,\,y_{5} (0) = 1 + \delta_{2} y_{6} (0),\,\,\,\,y_{7} (0) = 1 + \delta_{2} y_{7} (0) \\ & y_{2} (\infty ) \to 0,\,\,\,\,\,\,y_{5} (\infty ) \to 0,\,\,\,\,y_{7} (\infty ) \to 0 \\ \end{aligned}$$

### Physical quantities of interest

The $C_{f}$: skin friction coefficient, $Nu_{x}$: local Nusselt number, $Sh_{x}$: local Sherwood number are given as:

11 $$C_{f} = \frac{{\tau_{w} }}{{\rho u_{w}^{2} }};\,Nu_{x} = \frac{{xq_{w} }}{{\kappa \left( {T_{w} - T_{\infty } } \right)}};\;{\text{and}}\;Sh_{x} = \frac{{x\,J_{w} }}{{D_{m} (C_{w} - C_{\infty } )}}$$

where

12 $$\begin{aligned} & \tau_{w} = \mu \left. {\left( {\frac{\partial u}{{\partial y}}} \right)} \right|_{y = 0} , \, \;{\text{wall shearing stress}};\;q_{w} = \left. { - \left( {\kappa + \frac{{16\sigma_{1} T^{3} }}{3k^*}} \right)\left( {\frac{\partial T}{{\partial y}}} \right)} \right|_{y = 0} ,\;{\text{surface heat flux}}; \\ & J_{w} = \left. { - D_{m} \left( {\frac{\partial C}{{\partial y}}} \right)} \right|_{y = 0} ,\;{\text{ surface mass flux}} \\ \end{aligned}$$

Utilizing Eq. (6) within the context of Eq. (11), the subsequent expressions for skin friction coefficient ($C_{f}$), local Nusselt number ($Nu_{x}$) and local Sherwood number ($Sh_{x}$) are attained:

13 $$C_{f} \sqrt {{\text{Re}}_{x} } = f^{\prime\prime}\left( 0 \right),$$

14 $$Nu/\sqrt {{\text{Re}}_{x} } = - \left[ {1 + \frac{4R}{3}\left\{ {1 + (\theta_{w} - 1)\theta (0)} \right\}^{3} } \right]\theta ^{\prime}(0),$$

15 $$Sh/\sqrt {{\text{Re}}_{x} } = - \phi ^{\prime}(0).$$

${\text{Re}}_{x} = \frac{{bx^{2} }}{\upsilon }$ is local Reynold number.

## Result and discussion

The transfigured governing Eqs. (7–9) mentioned earlier are numerically addressed using bvp4c function, a built-in MATLAB tool, with the boundary conditions specified in Eq. (10). The outcomes thus obtained signify significant implications of non-dimensional governing variables as $\beta_{1}$ and $\beta_{2}$ that represents Deborah number in relaxation and retardation time respectively, $M$ magnetic field parameter, $\delta_{1} ,\,\delta_{2} ,\,\delta_{3}$ Velocity, temperature and mass slip parameter, $Kp$ porous medium parameter, $R$ radiation parameter, temperature ratio parameter $\theta_{w}$, $Me$ Melting parameter, $Ec$ Eckert number, Inclined angle $\alpha$, $Sc$ Schmidt number, parameter of Chemical reaction $Kn$, non-uniform heat source/sink parameters $A^{*}$ on velocity, temperature, and concentration profile respectively. Mathematical computations are conducted, and the favourable effects of the parameters involved are investigated concerning the profiles of velocity, temperature, and mass, utilizing relevant graphical data. To validate the current findings, the results are compared with those of Anderson et al.^27^, Prasad et al.^28^, Khan et al.^29^ and Wang^26^, which are presented in Tables 1 and 2. The observed concordance with these works is noted as satisfactory and supportive Table 3 show the $C_{f}$. $Nh_{x}$, $Sh_{x}$ for various value of the physical parameter.

**Table 1:** Comparison of $- \theta ^{\prime}(0)$ for distinct values of Pr when $\beta_{1}$ =   $\mathop A\nolimits^{*}$ =   $\beta_{2}$ = $S$ =   $R$ =   $M$ =   $\mathop B\nolimits^{*}$ =   $Kn$ =  $\alpha$ = 0.

$\Pr$	Wang^26^	Khan et al.^29^	Present study
0.7	0.454	0.454	0.4540471
2.0	0.911	0.911	0.9113625

**Table 2:** Comparison of $- f^{\prime\prime}(0)$ for distinct values of $M$ when $\beta_{1}$ = $\beta_{2}$ = $S$ = $R$ = $Ec$ = $\alpha$ = $\mathop A\nolimits^{*}$ = $\mathop B\nolimits^{*}$ = $Kn$ = 0.

$M$	Anderson et al.^27^	Prasad et al.^28^	Present study
1.0	1.414000	1.414449	1.4142586
1.5	1.581000	1.581139	1.5811481
2.0	1.732000	1.732203	1.7320505

**Table 3:** Show the values of the skin friction coefficient, local Sherwood number and Nusselt number for various parameters.

$M$	$\Pr$	$Kp$	$Me$	$S$	$R$	$\beta_{1}$	$\beta_{2}$	$Cf$	$- Nh_{x}$	$- Sh_{x}$
3								− 2.005549569	0.645206952	1.077520268
6								− 2.296626507	0.506509076	1.034232813
9								− 2.530221494	0.405747366	1.004277722
	2							− 2.000664821	0.330581757	0.978790757
	4							− 2.008165049	0.918660005	1.128786293
	6							− 2.010855432	1.363926371	1.180321909
		3						− 2.005549569	0.645206952	1.077520268
		4						− 2.115899795	0.623176740	1.060715986
		5						− 2.216728742	0.603089981	1.046201739
			0.02					− 1.890180743	0.804089308	1.193364385
			0.04					− 1.883386033	0.669357234	1.097208984
			0.06					− 1.876809918	0.529722956	1.003258477
				0.1				− 1.835850688	0.520687468	1.307160416
				0.3				− 1.894672617	0.888273369	1.255930937
				0.5				− 1.920321937	1.308675802	1.591665706
					0.1			− 1.920321937	1.308675802	1.591665706
					0.2			− 1.920318186	1.306584930	1.591619476
					0.3			− 1.920314470	1.304514156	1.591573688
						0.4		− 1.884458040	1.312659296	1.598132897
						0.6		− 1.860668943	1.315236719	1.602552871
						0.8		− 1.837154418	1.317709875	1.607029985
							0.5	− 1.598228442	1.332055766	1.637200363
							0.7	− 1.497316747	1.337037670	1.649842234
							0.9	− 1.416447321	1.339893294	1.659941049

**Figure 2:**
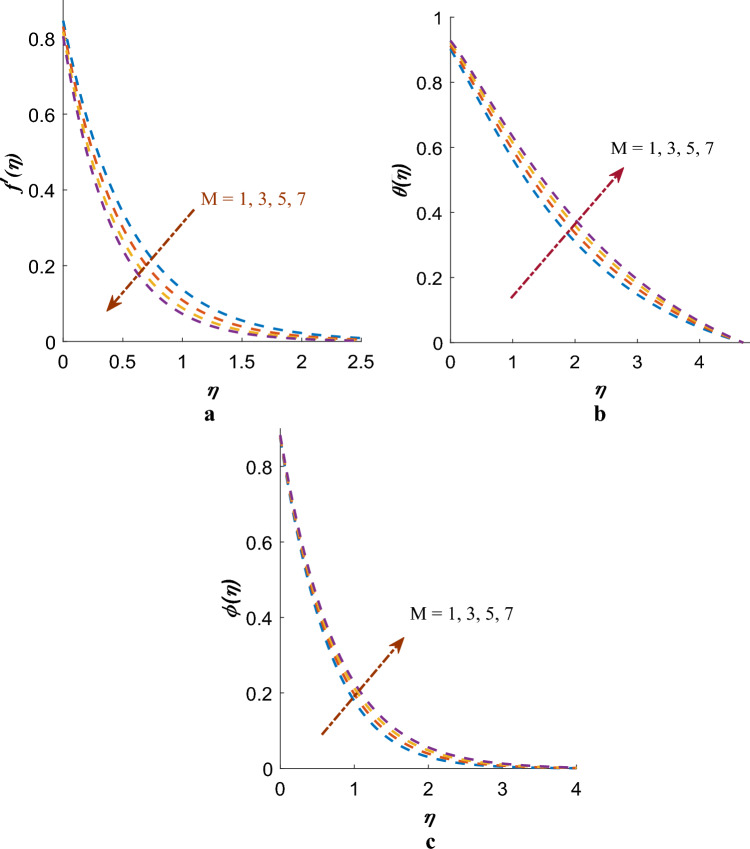
(**a**) Variation of $M$ on $f^{\prime}\left( \eta \right)$. (**b**) Effect of $M$ on $\theta \left( \eta \right)$. (**c**) Variation of $M$ on $\phi \left( \eta \right)$.

Figure 2a–c depict consequence of magnetic-field factor ($M$) on velocity, temperature and concentration profiles respectively. It has been observed that velocity profile goes down by enhancing $M$ (see Fig. 2a). Physically, when a magnetic field is applied to a conducting fluid, it induces an electric current within the fluid. The interaction of this electric current with the magnetic field results in a force referred to as the Lorentz force. As the magnetic field strength increases, Lorentz force becomes more pronounced, creating greater resistance to fluid motion and consequently leading to a reduction in fluid velocity. However, contrasting effects are noticed on the profiles of $\phi \left( \eta \right)$, $\theta \left( \eta \right)$ (see Fig. 2b,c).

**Figure 3:**
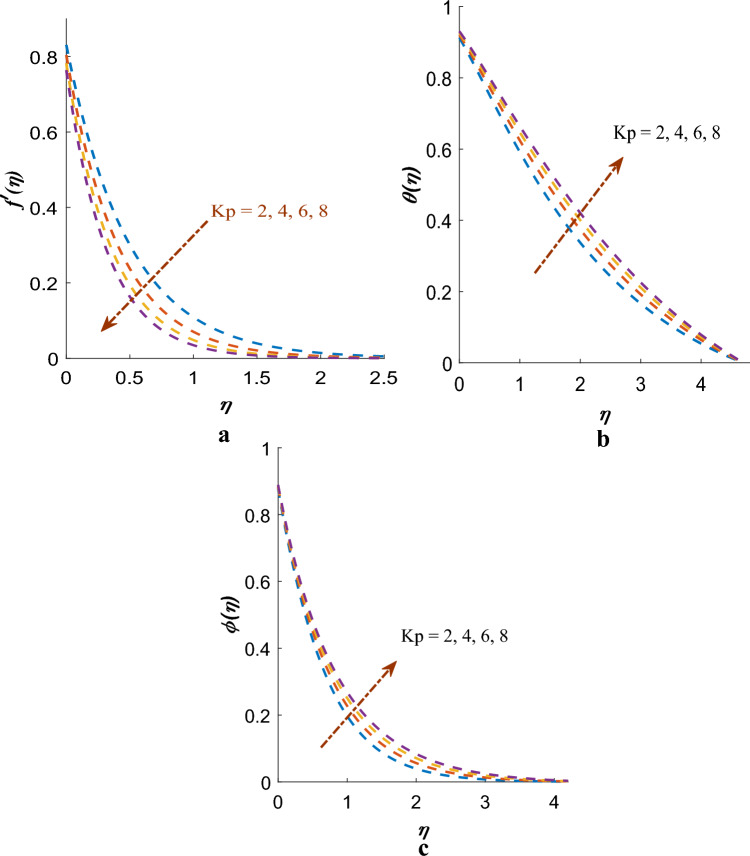
(**a**) Effect of $Kp$ on $f^{\prime}\left( \eta \right)$. (**b**) Effect of $Kp$ on the $\theta \left( \eta \right)$. (**c**) Effect of $Kp$ on $\phi \left( \eta \right)$.

The velocity ($f^{\prime}\left( \eta \right)$), temperature ($\theta \left( \eta \right)$), concentration ($\phi \left( \eta \right)$) profile are influenced by the porous medium parameter $\left( {Kp} \right)$, as illustrated in Fig.  3a–c. The thickness of temperature and concentration profiles are extended as $Kp$ upsurges while the $f^{\prime}\left( \eta \right)$ profile gets diminished. Figure 3a illustrates that elevating values of $Kp$ results in a reduction in flow velocity. or lessening permeability parameter $\left( {\mathop k\nolimits_{p} } \right)$. Momentum equation reveals that Darcian resistance force is inversely proportional to the $\left( {k_{p} } \right)$. Consequently, a low permeability will produce a significant Darcian resistance to fluid flow. Thus, flow-field is observed diminishing as the $Kp$ increases.

**Figure 4:**
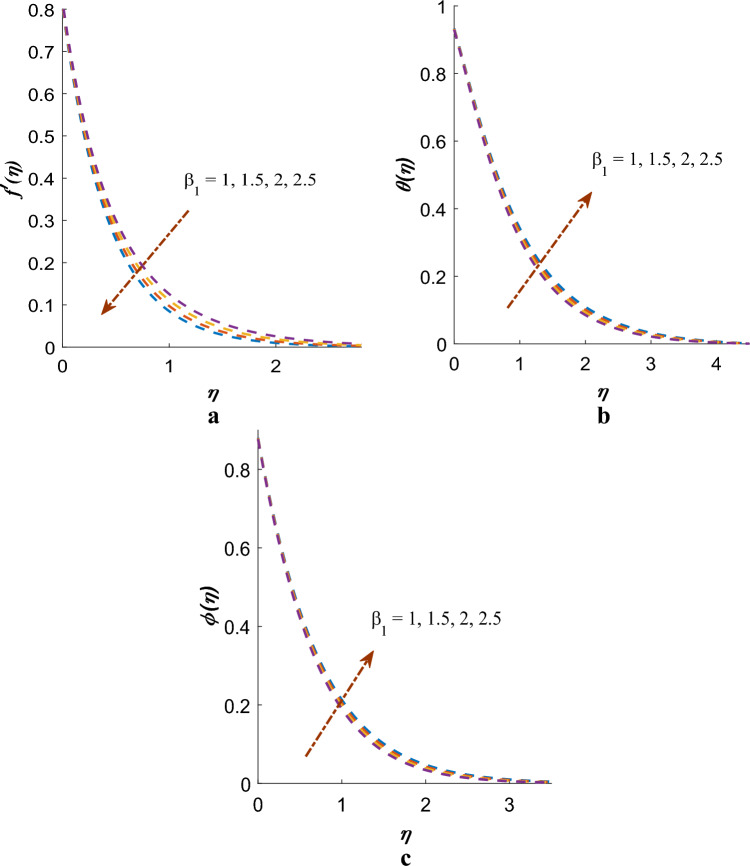
(**a**) Effect of $\beta_{1}$ on $f^{\prime}\left( \eta \right)$. (**b**) Effect of $\beta_{1}$ on $\theta \left( \eta \right)$. (**c**) Effect of $\beta_{1}$ on $\phi \left( \eta \right)$.

**Figure 5:**
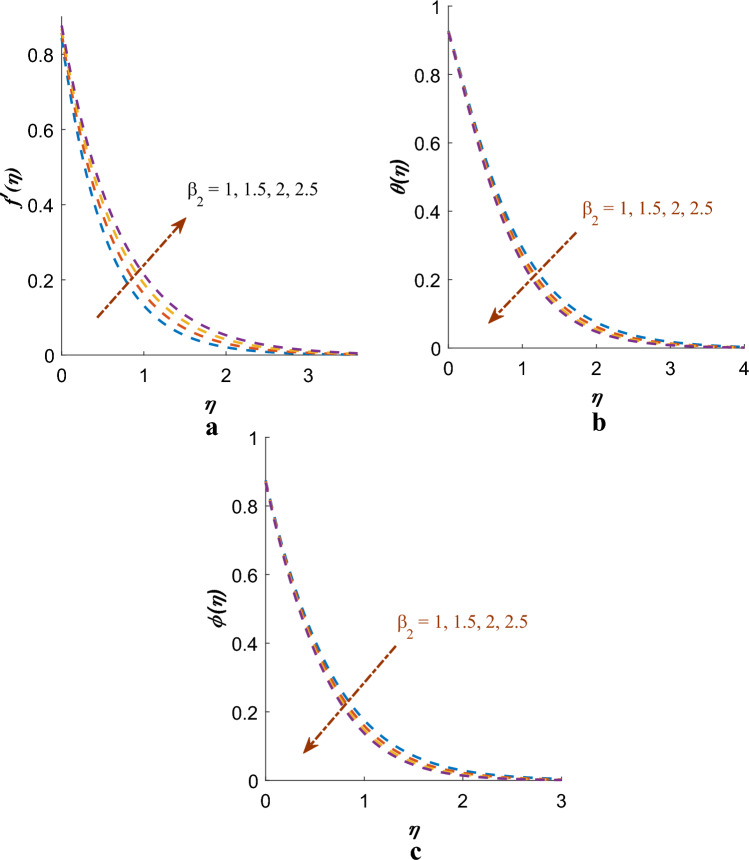
(**a**) Effect of $\beta_{2}$ on $f^{\prime}\left( \eta \right)$. (**b**) Effect of $\beta_{2}$ on $\theta \left( \eta \right)$. (**c**) Effect of $\beta_{2}$ on $\phi \left( \eta \right)$.

Figure 4a–c depict the interesting phenomenon on Deborah number ($\beta_{1}$) on the profiles of velocity, temperature, and mass. The velocity distribution diminutions with growing values of the Deborah number, while the thickness of temperature and mass boundary layers increases. In physical terms, the ratio of relaxation time to observation time is linked to the Deborah number. As an increase in the Deborah number indicates a longer relaxation time, leading to greater resistance to the fluid motion and, consequently, a decrease in the velocity profile. Figure 5a–c are shows $f^{\prime}\left( \eta \right)$, $\theta \left( \eta \right)$, $\phi \left( \eta \right)$ distribution affected by Deborah number $\left( {\beta_{2} } \right)$ (in the terms of retardation time). It has been established that the velocity profile shows notable improvement with higher values of the Deborah number, while the temperature and concentration profiles are observed to decrease.

**Figure 6:**
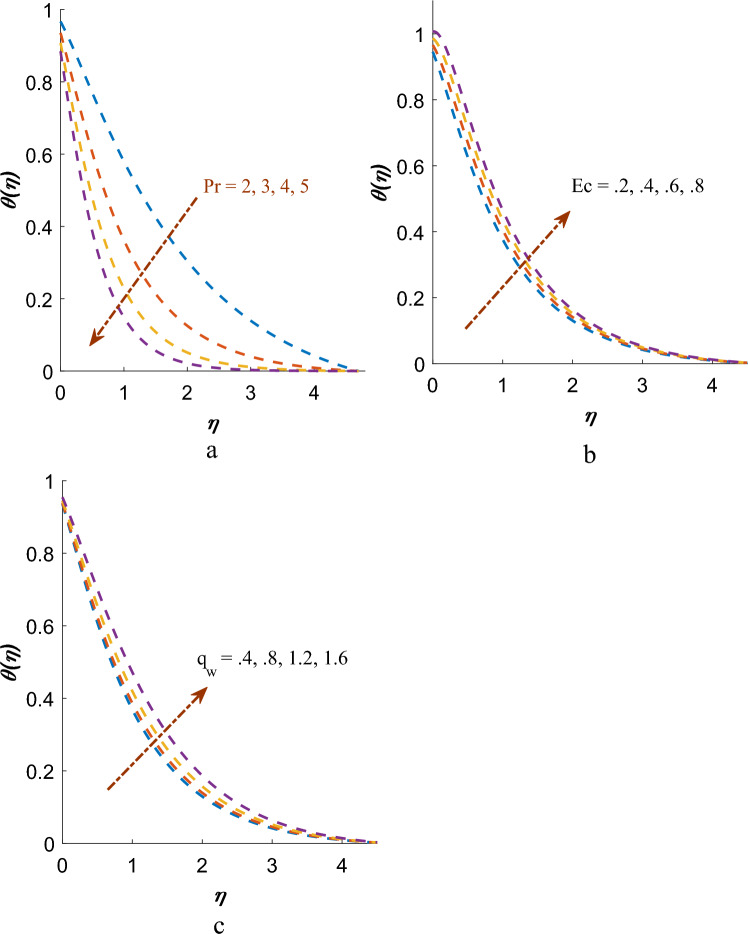
(**a**) Effect of $\Pr$ on $\theta \left( \eta \right)$. (**b**) Effect of $Ec$ on $\theta \left( \eta \right)$. (**c**) Effect of $\theta_{w}$ on $\theta \left( \eta \right)$.

**Figure 7:**
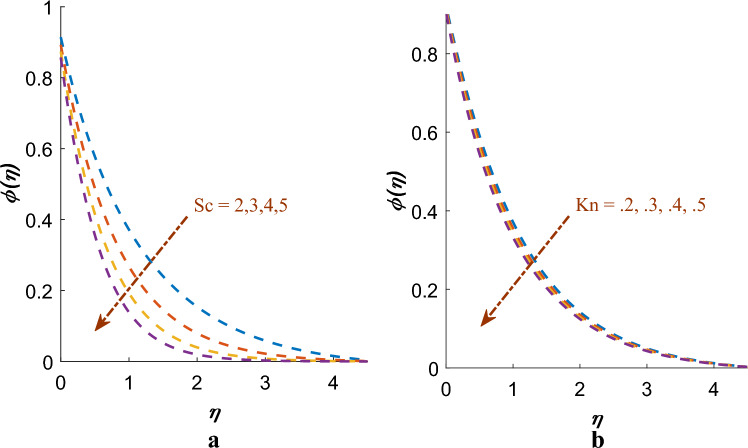
(**a**) Effect of $Sc$ on $\phi \left( \eta \right)$. (**b**) Effect of $Kn$ on $\phi \left( \eta \right)$.

Impact of $\Pr$, $Ec$ and $\theta_{w}$ on temperature $\theta \left( \eta \right)$ profiles are constituted in Fig. 6a-c. It has been concluded that temperature distribution decreases for $\Pr$ whereas temperature distribution improves for $Ec$ and $\theta_{w}$. Impact of $Sc$ and $Kn$ are instituted in Fig. 7a,b on $\phi \left( \eta \right)$ profiles. Mass profile decreases with rising values $Sc$ and $Kn$. Physically, an increase in the $Sc$ demonstrates an enhancement in the momentum diffusivity of the fluid, demonstrating increase rate of mass flow important to fewer mass credits on surface.

**Figure 8:**
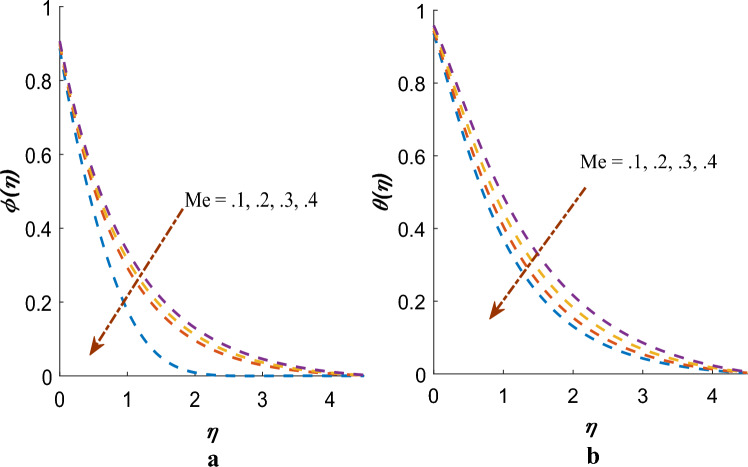
(**a**) Effect of $Me$ on $\phi \left( \eta \right)$. (**b**) Effect of $Me$ on $\theta \left( \eta \right)$.

Figure 8a,b indicates that concentration profile $\phi \left( \eta \right)$ and temperature $\theta \left( \eta \right)$ are declined when the physical parameter $Me$ is increased accordingly. Physically, when the melting parameter increases, it implies that the heat generation due to melting becomes more significant. As a result, more heat is generated within the boundary layer close to the sheet surface. This increased heat generation counteracts the convective heat transfer from the fluid flow. Consequently, the temperature profile in the BL decreases as heat generation due to melting dominates the heat transfer process.

**Figure 9:**
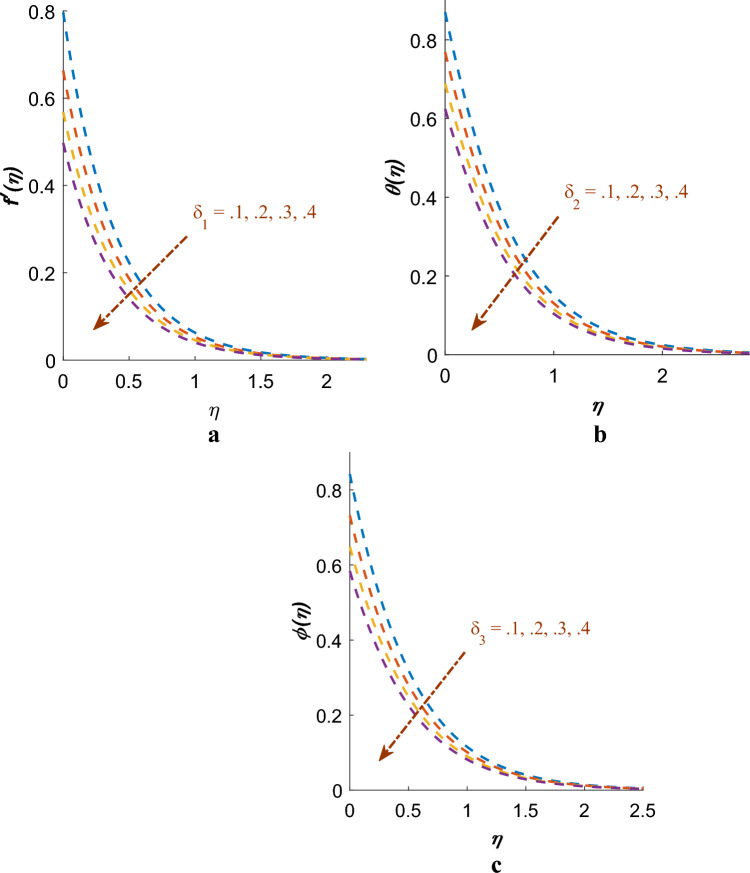
(**a**) Effect of $\delta_{1}$ on $f^{\prime}\left( \eta \right)$. (**b**) Effect of $\delta_{2}$ on $\theta \left( \eta \right)$. (**c**) Effect of $\delta_{3}$ on $\phi \left( \eta \right)$.

Figure 9a–c serve to illustrate the effects $\delta_{1}$, $\delta_{2}$ and $\delta_{3}$ on $f^{\prime}\left( \eta \right)$, $\theta \left( \eta \right)$, $\phi \left( \eta \right)$ profile. It has been concluded that $f^{\prime}\left( \eta \right)$, $\theta \left( \eta \right)$ and $\phi \left( \eta \right)$ profiles is commendably declined for higher values of $\delta_{1}$, $\delta_{2}$ and $\delta_{3}$. Physically, as the velocity slip parameter increases, it indicates a larger slip velocity at the fluid–solid interface, where the fluid molecules experience reduced interaction with the solid surface. As a result, the momentum transfer among the fluid and the solid surface weakens, leading to a lessening in the velocity profile.

**Figure 10:**
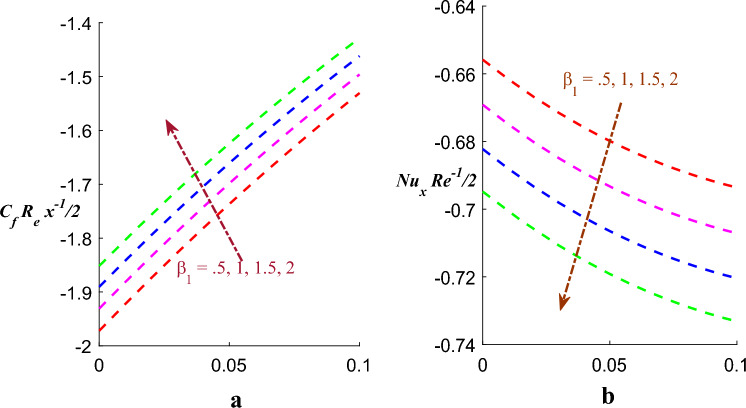
(**a**) Effect of $\beta_{1}$ on $Cf$. (**b**) Effect of $\beta_{1}$ on $Nu$.

**Figure 11:**
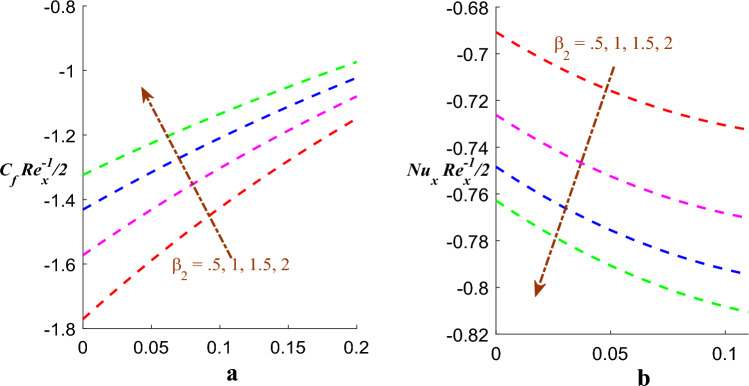
(**a**) Effect of $\beta_{2}$ on $Cf$. (**b**) Effect of $\beta_{2}$ on $Nu$.

Figure 10a,b exhibits nature of $\left( {\beta_{1} } \right)$ on skin frication $\left( {Cf_{x} } \right)$ and Nusselt number $\left( {Nu_{x} } \right)$. It is found that skin frication increases and on the other side Nusselt number decreases for the value of $\beta_{1}$ whereas Fig. 11a,b exhibits consequences of $\left( {\beta_{2} } \right)$ on skin frication $\left( {Cf_{x} } \right)$ and Nusselt number $\left( {Nu_{x} } \right)$. It is found that $\left( {Cf_{x} } \right)$ increases whereas $\left( {Nu_{x} } \right)$ is subside for value of $\beta_{2}$.

## Conclusions

The present study offers numerical results for the magnetohydrodynamic flow of an Oldroyd-B fluid over a continuously stretching sheet. The numerical outcomes of the transformed ODE’s are presented graphically, allowing for an exploration of the acceptable values of the regulating parameters in the specified problem. The essential outcomes are listed as follows:

- An increase in the porous medium parameter $Kp$ leads to a reduction in the corresponding velocity profile $f^{\prime}\left( \eta \right)$.
- An elevation in the $\beta_{1}$ is associated with a decline in the corresponding thevelocity profile $f^{\prime}\left( \eta \right)$. On the flip side, an enhancement in the Deborah number results in a rise in the temperature profile.
- The velocity profile is positively influenced by the Deborah number $\beta_{2}$, indicating that velocity is an increasing function dependent on the effects of the Deborah number $\beta_{2}$.
- $\theta \left( \eta \right)$ profile is decreasing as a functions of corresponding parndtl number $\Pr$.
- The concentration field diminishes as the corresponding Schmidt number $Sc$ increases.

## Data Availability

The datasets used and/or analysed during the current study available from the corresponding author on reasonable request.

## List of symbols

$$b$$: Constant parameter
$B_{0}$: Strength of magnetic field (kgs^−^^2^A^−^^1^)
$C$: Fluid’s concentration (kgm^−^^3^)
$C_{f}$: Skin friction coefficient
$C_{p}$: Specific heat (J kg^−^^1^ k^−^^1^)
$C_{w}$: Fluid concentration at the wall (kgm^−^^3^)
$D_{m}$: Coefficient of mass diffusion (m^2^s^−^^1^)
$$Ec$$: Eckert number
$$\kappa$$: Thermal conductivity (W/m K)
$k^{*}$: Mean absorption coefficient
$K_{n}$: Chemical reaction parameter
$L_{1}$: Velocity slip factor
$L_{2} ,\,L_{3}$: Temperature and Mass slip factor
$$K_{p}$$: Porous parameter
$M$: Magnetic field parameter
$$Me$$: Melting parameter
$Nu_{x}$: Local Nusselt number
$$\Pr$$: Prandtl number
$q_{w} (x)$: Local surface heat flux (W m^−^^2^)
$q_{r}$: Radiative heat flux
$q^{*}$: Heat generation or absorption
${\text{Re}}_{w}$: Local Reynolds number
$$R$$: Radiation parameter
$A^{*} ,\,B^{*}$: Space dependent parameter
S: Section/injection parameter
$$Sh_{x}$$: Sherwood number
$T_{0}$: Solid surface temperature (K)
$T_{\infty }$: Free stream temperature (K)
$u_{w}$: Surface velocity (ms^−^^1^)
$u,\,v$: Velocity component corresponding to horizontal and the vertical direction (ms^−^^1^)
$\rho$: Fluid density (kgm^−^^1^)
$$\upsilon$$: Kinematic viscosity (m^2^s^−^^1^)
$\sigma$: Electrical conductivity (sm^−^^1^)
$\alpha$: Angle of inclination of magnetic field
$$\lambda_{1}$$: Relaxation times
$$\lambda_{2}$$: Retardation times
$$\delta_{1}$$: Velocity slip
$$\delta_{2}$$: Temperature slip
$$\delta_{3}$$: Mass slip
$\beta_{1}$: Deborah number in relaxation time
$\beta_{2}$: Deborah number in retardation time
$\theta_{w}$: Temperature ratio parameter
$f^{\prime}\left( \eta \right)$: Non-dimensional velocity parameter
$\theta \left( \eta \right)$: Non-dimensional temperature parameter
BL: Boundary layer
$\phi \left( \eta \right)$: Non-dimensional concentration parameter
